# Exploring psilocybin-assisted psychotherapy in the treatment of methamphetamine use disorder

**DOI:** 10.3389/fpsyt.2023.1123424

**Published:** 2023-03-14

**Authors:** Jonathan Brett, Elizabeth Knock, P. Todd Korthuis, Paul Liknaitzky, Kevin S. Murnane, Christopher R. Nicholas, James C. Patterson, Christopher S. Stauffer

**Affiliations:** ^1^Department of Clinical Pharmacology, St. Vincent’s Hospital, Sydney, NSW, Australia; ^2^School of Population Health, Medicines Intelligence Centre of Research Excellence, University of New South Wales, Sydney, NSW, Australia; ^3^Alcohol and Drug Service, St. Vincent’s Hospital, Sydney, NSW, Australia; ^4^Section of Addiction Medicine, Oregon Health & Science University, Portland, OR, United States; ^5^Department of Psychiatry, School of Clinical Sciences, Monash University, Caulfield, VIC, Australia; ^6^Turner Institute for Brain and Mental Health, School of Psychological Sciences, Monash University, Caulfield, VIC, Australia; ^7^Louisiana Addiction Research Center, Department of Pharmacology, Toxicology & Neuroscience, Shreveport, LA, United States; ^8^Department of Psychiatry and Behavioral Medicine, Louisiana State University Health, Shreveport, LA, United States; ^9^Department of Family Medicine and Community Health, University of Wisconsin-Madison, Madison, WI, United States; ^10^Department of Mental Health, Veterans Affairs Portland Health Care System, Portland, OR, United States; ^11^Social Neuroscience and Psychotherapy Lab, Department of Psychiatry, Oregon Health and Science University, Portland, OR, United States

**Keywords:** methamphetamine use disorder, psilocybin-assisted psychotherapy, Addiction Medicine, stimulant use disorder, methamphetamine, psilocybin

## Abstract

Methamphetamine use disorder is a chronic relapsing condition associated with substantial mental, physical, and social harms and increasing rates of mortality. Contingency management and psychotherapy interventions are the mainstays of treatment but are modestly effective with high relapse rates, while pharmacological treatments have shown little to no efficacy. Psilocybin-assisted psychotherapy is emerging as a promising treatment for a range of difficult-to-treat conditions, including substance use disorders; however, no studies have yet been published looking at psilocybin-assisted psychotherapy in the treatment of methamphetamine use disorder. Here we review the rationale for psilocybin-assisted psychotherapy as a potential treatment for this indication, and describe practical considerations based on our early experience designing and implementing four separate clinical trials of psilocybin-assisted psychotherapy for methamphetamine use disorder.

## 1. Introduction

Methamphetamine is a highly addictive psychostimulant with evidence of neurotoxic properties (1–3) and is consistently ranked as one of the most harmful illicit substances—both to the person using and to society (4, 5). Methamphetamine use disorder is a chronic relapsing condition increasingly associated with harms that include mental and physical illness, intimate partner violence, family disruption, health care system pressures, homelessness, crime, and mortality (6–10). At present, there are no approved medications to treat methamphetamine use disorder, despite a large body of research investigating potential pharmacological interventions (10–12). The most effective non-pharmacological evidence-based intervention for the management of methamphetamine use disorder is contingency management, a non-psychotherapy behavioral approach that most often involves monetary-based reinforcement for drug-negative urine specimens (13, 14). In practice, psychotherapy is often the standard of care given resource limitations in real world settings, including cognitive behavioral therapy and motivational interviewing. Multiple barriers to treatment exist, such as stigmatizing experiences within the health care system and existing treatment options not meeting patient needs (15). Moreover, people who use methamphetamine consistently demonstrate more challenges in treatment and recovery compared to those using other substances (16, 17). A recent systematic review estimated methamphetamine treatment drop-out rates to be 53.5% (95% CI: 16.5, 87.0), the highest compared to other substances, including the psychostimulant cocaine, with the average drop-out across all substances being 30.4% (95% CI: 27.2-33.8) (18). Lastly, rapid return to use is the norm (19). Innovative and effective treatments are urgently needed to address the mounting methamphetamine epidemic (20, 21).

Psilocybin is a non-addictive classic psychedelic (22, 23) with neuroplasticity-inducing properties (24, 25). It is consistently ranked as one of the least harmful illicit psychoactive substances (4, 5), despite being among the most highly regulated substances globally. Psilocybin is a tryptamine alkaloid with serotonin 2A receptor (5-HT_2*A*_) agonism (26)—and complex pharmacology at many additional serotonin and non-serotonin receptors—that induces marked transitory changes in perception, cognition, and affect. Early phase clinical trials demonstrate that one to three moderate-to-high doses (20 mg to 30 mg/70 kg or more) of psilocybin combined with a brief course of psychotherapy (i.e., psilocybin-assisted psychotherapy) is safe, feasible, and preliminarily efficacious at alleviating symptoms of major depressive disorder, anxiety and depression associated with end-of-life diagnoses, and alcohol and tobacco use disorder (27–30). Psilocybin-assisted psychotherapy received a ‘Breakthrough Therapy’ designation from the United States Food and Drug Administration for both major depressive disorder and treatment-resistant depression. This designation was created to expedite the development and review of drugs intended to treat serious or life-threatening conditions for which preliminary evidence suggests substantial improvement over available options.

This group of authors represents investigators on four separate registered or soon-to-be registered trials exploring the safety, feasibility, and preliminary efficacy of psilocybin-assisted psychotherapy for methamphetamine use disorder across the United States (ClinicalTrials.gov Identifiers: NCT04982796 and NCT05322954) and Australia (ANZCTR: ACTRN12622000463774). In this manuscript, we review the rationale for psilocybin-assisted psychotherapy to treat methamphetamine use disorder and consider putative mechanisms. We explore potential challenges and practical considerations specific to this treatment modality and clinical population.

## 2. Rationale for psilocybin-assisted psychotherapy in the treatment of methamphetamine use disorder

Early phase clinical trials of psilocybin-assisted psychotherapy have demonstrated promising results for treatment of other substance use disorders. For example, psilocybin-assisted psychotherapy for alcohol use disorder achieved substantial reductions in alcohol use in a small (*n* = 10), open-label, proof-of-concept study (31) and a larger (*n* = 93), double-blind trial that randomized participants to two psilocybin sessions versus an active placebo (diphenhydramine) control along with psychotherapy in both groups (30). The latter demonstrated a mean absolute difference of 13.9% (95% CI: 3.0, 24.7%) between treatment groups in percentage of heavy drinking days during the 32-week follow-up period. An open-label trial of psilocybin-assisted psychotherapy for tobacco smoking cessation (*n* = 15) resulted in 80% of participants demonstrating abstinence at 6 months (32) and 67% at 12 months (33). A multisite, randomized, controlled trial of psilocybin-assisted psychotherapy for tobacco smoking cessation is currently underway, partially funded by the United States National Institute on Drug Abuse (NIDA) (ClinicalTrials.gov Identifier: NCT05452772). Finally, preliminary reports (34) from a randomized controlled trial of psilocybin-assisted psychotherapy for cocaine use disorder are promising (NCT02037126). While there are not yet any data from clinical trials of psilocybin-assisted psychotherapy for methamphetamine use disorder, cross-sectional survey data show that naturalistic use of psilocybin and lysergic acid diethylamide (LSD) — another classic psychedelic — has been associated with reductions in stimulant craving and use (35).

There is an emerging hypothesis that psychedelic-assisted psychotherapy holds transdiagnostic treatment potential, possibly due to its ability to increase neuronal and mental plasticity (36). As such, evidence suggests that psilocybin-assisted psychotherapy could address various psychiatric conditions commonly comorbid with methamphetamine use (27), such as treatment-resistant depression and anxiety (37, 38). There are no adequate treatments for dual diagnoses of methamphetamine use disorder and psychiatric illness; although such comorbidity adversely affects methamphetamine treatment outcomes (39, 40), and treatments of higher intensity are recommended (41). Treatment planning can be complicated by a lack of initial clarity around whether the comorbid psychiatric illness was pre-existing, and possibly contributed to methamphetamine use, or if it is secondary to prolonged use, acute intoxication, or acute withdrawal from methamphetamine. If pre-existing, treatment of the comorbid psychiatric illness is likely required in addition to treatment for methamphetamine use disorder; if secondary, symptoms may clear over time with methamphetamine abstinence. The transdiagnostic treatment potential of psilocybin-assisted psychotherapy may help simplify treatment planning in a dual diagnosis treatment setting, thus preventing adverse outcomes related to the high rates of attrition and return to methamphetamine use commonly seen among people seeking treatment (19, 42).

Psilocybin-assisted psychotherapy may also target more fundamental and common drivers of psychopathology than diagnostic syndromes (36, 43, 44). One leading theory to explain psilocybin’s therapeutic potential posits that classic psychedelics reduce the precision-weighting (confidence) of predictive models supporting maladaptive beliefs and behaviors—in effect, increasing a psychologically receptive state to view life challenges, and perhaps substance use, from new perspectives (36, 45). This may be partially supported by the observed changes in connectivity of the default mode network and various other executive control networks in the brain following psilocybin dosing that may remediate changes observed in people with substance use disorders (46, 47). Indeed, decoupling of the default mode network through other pharmaceutical interventions is associated with reduction in risky decision-making among patients with methamphetamine use disorder (48). Another hypothesized mechanism is psilocybin-induced neuroplasticity (49). Drugs with high addiction potential, like methamphetamine, can hijack neuroplastic mechanisms in key brain regions, such as the mesolimbic dopamine system, ultimately contributing to the maladaptive maintenance of addictive behavior (50). Conversely, preclinical evidence suggests that psilocybin may induce neuroplasticity in the prefrontal cortex and hippocampus (24, 25), which could potentially be leveraged in the context of psychotherapy to facilitate extinction learning, long-term alterations in emotion processing and reward and stress processing, and increased psychological flexibility (49, 51–53).

Methamphetamine use has been inversely associated with perceived social support (54), and the most common barriers to accessing treatment for people with methamphetamine use disorder tend to be psychosocial in nature (15). Social isolation and loneliness are common among methamphetamine users and associated with poor methamphetamine treatment outcomes (55, 56). In contrast, within a supportive context, psilocybin increases emotional empathy, attachment security, connectedness, and other pro-social effects (57–59).

Lastly, psilocybin can occasion profound mystical-type experiences that mediate ratings of substantial personal meaning and spiritual significance (57, 60, 61). The participant-reported strength of the psilocybin-occasioned mystical-type experience has also been strongly associated with reductions in alcohol use and tobacco cessation in clinical trials of psilocybin-assisted psychotherapy for those indications (31, 62). Spiritual engagement is a common recovery strategy used by individuals with methamphetamine use disorder (63), and an experience of spiritual awakening among individuals in Narcotics Anonymous (*N* = 527) was associated with lower rates of drug craving (64). While Narcotics Anonymous is traditionally an abstinence-based program, incorporating psychedelics into a 12-step model for addiction is not a new idea (65). Elucidating the respective contributions of various potential mechanisms of action should be a focus of future research and can ultimately contribute to more targeted interventions.

## 3. Practical considerations

The design and implementation of clinical trials to test the safety, feasibility, and preliminary efficacy of psilocybin-assisted psychotherapy for methamphetamine use disorder merits several considerations unique to this population.

### 3.1. Balancing safety with access

Eligibility criteria may need to be adjusted to address safety concerns salient to methamphetamine use. For example, the prevalence of stimulant-induced psychosis among people who use methamphetamine ranges from 37 to 43% (66). As is common practice in research with methamphetamine users, it is advisable in psilocybin trials to exclude people with a primary psychotic disorder or first-degree relatives with a primary psychotic disorder. Further exclusions might be considered within early proof-of-concept trials based on additional risk factors for first-break psychosis–such as being under 25 years old, active self-harm, or a history of violent behavior (67, 68)—or endophenotypes for psychotic illness (69). However, investigators must balance safety with real-world applicability and treatment access when considering clinical trial design for this indication. As the neurobiological correlates of primary psychotic disorders and stimulant-induced psychosis are thought to be quite distinct (70), so too may be the psilocybin-related risk for psychosis and stimulant-induced psychosis. The question of optimal timing to begin psilocybin-assisted psychotherapy relative to last use of methamphetamine also remains to be answered and may vary based on factors such as treatment setting, addiction severity, and comorbid diagnoses. Early phase studies can base eligibility criteria on aspects of the set and setting that affect both safety and access. For example, in a context that offers substantial support (e.g., within a residential treatment facility with experienced psychedelic facilitators), psilocybin trials might allow enrolment of participants who have a history of stimulant-induced psychosis (but without a primary psychotic disorder), more severe methamphetamine use disorder, or comorbid psychiatric disorders in order to serve as a more pragmatic design with greater generalizability (71).

Polysubstance use is prevalent among people who use methamphetamine (37, 72); those with polysubstance use and greater use severity may stand to benefit most from innovative transdiagnostic treatments. Clinical trials must also weigh inclusion of participants taking psychiatric medications. Historically, taking concomitant antidepressants has been an exclusion criterion due to concerns about safety or psilocybin-blunting drug-drug interactions (73). However, excluding participants taking psychiatric medications introduces significant bias and reduces generalizability, possibly limiting access in the future. Moreover, stopping psychiatric medications to achieve clinical trial eligibility introduces the risk of symptom re-emergence (e.g., suicidality) and medication withdrawal syndromes. Studies to-date looking at the impact of serotonergic antidepressants on the intensity of psilocybin effects have demonstrated mixed results (73, 74). Future trials might consider including those with comorbid substance use disorders and assess whether psilocybin therapies can be co-administered alongside common psychotropic medications without increasing safety concerns or compromising clinical benefit.

### 3.2. Patient representation

Methamphetamine use disorder has increased disproportionately among marginalized communities. For instance, there has been a 10-fold increase in methamphetamine use among African American individuals between 2015 and 2019, compared to a 3-fold increase among White individuals (9). Similarly, Aboriginal Australians are more than twice as likely than non-Aboriginal Australians to use methamphetamine (75). Other risk factors for methamphetamine use and methamphetamine use disorder include lower educational attainment, lower annual household income, lack of insurance, housing instability, criminal justice involvement, and medical and psychiatric comorbidities (9). To explore the utility of this treatment approach for individuals most in need, psychedelic study samples should represent diverse populations and actively address health disparities known to exist within psychedelic therapy research (76). Funding for transportation and, potentially, phones would help support community-based outpatient protocols. As part of improving representation within trials, members of marginalized communities might be invited to contribute to protocol design and implementation. How these studies can be extended to those with marginalized identities and other underrepresented groups, who may bear a disproportionate burden of methamphetamine, needs to be considered.

### 3.3. Dosage

Another concern, though theoretical, involves the potential for blunting of psilocybin’s psychedelic effect among people who use methamphetamine. Dose blunting was flagged as a concern in a recent Phase II study of psilocybin-assisted psychotherapy for alcohol use disorder. In this study, the second dose of psilocybin was escalated to 40 mg/70 kg in 30% of participants due to a low subjective measure of psychedelic experience (Mystical Effects Questionnaire score < 0.6) following a 25 mg/70 kg first dose (30). There are putative concerns specific to methamphetamine; the predominant mechanism of action of methamphetamine is to elevate the concentration of the monoamines dopamine and norepinephrine within the synaptic cleft through substrate-based release and interrupted reuptake (77, 78). There are studies showing that crosstalk between the dopamine and noradrenergic systems with the serotonin system can lead to significant changes in serotonin transporters and receptors (79–82). This could modify the response to psilocybin, which, via its active metabolite psilocin, acts at 5-HT_2*A*_ receptors and other 5-HT receptors. However, this has not been investigated. Moreover, no trial to-date has investigated more than three dosing sessions of psilocybin for substance use disorders. Conversely, lower doses may be favored in the treatment of methamphetamine use disorder given a number of factors, including risks related to substance-induced psychosis, though this would contradict the positive dose-response trend in the literature related to treatment of other substance use and psychiatric disorders with psilocybin-assisted psychotherapy (61, 83). The optimal dosage of psilocybin in people with methamphetamine use disorder remains to be determined. Early studies that employ a dose-finding design could be beneficial; for instance, one of our registered trials includes a 25 mg psilocybin dose followed by an optional dose increase to 50 mg during a separate dosing session (NCT05322954).

### 3.4. Outcome measures

Rigorously measuring safety and feasibility outcomes in these early trials is important. Additionally, careful selection of primary clinical outcome measures is essential to determine preliminary effect sizes for later phase trials. Outcome measures must both satisfy the needs of agencies such as the United States Food and Drug Administration and the Australian Therapeutic Goods Administration, responsible for the safe approval of new medications, and be relevant to participants and their methamphetamine use patterns. Historically, trials have used abstinence as a primary endpoint. However, patients often report more alignment with harm reduction outcomes, including reductions in drug use, improvements in quality of life, and treatment satisfaction (84). Again, this emphasizes the importance of inviting feedback from the clinical population when constructing such studies.

For a treatment approach that may be associated with changes across a wide range of clinical symptoms, psilocybin-assisted psychotherapy studies should measure diverse secondary clinical outcomes such as motivation and self-efficacy for change (85), drug craving, symptoms of comorbid disorders (e.g., depression, anxiety, PTSD), level of disability, quality of life, and general well-being. Previous trials of psilocybin-assisted psychotherapy for other indications have identified measures of peak psychedelic experience (e.g., strength of the mystical-type experience) as correlated with or mediators of treatment response (31, 62, 86). Methamphetamine use disorder trials ought to explore these potential predictors too, alongside others, including brain network connectivity, attachment security (59), connectedness (87), psychological flexibility (88), and stress biomarkers such as inflammation, cortisol, and heart rate variability. Moreover, to determine more robust signals, early small trials might attempt to harmonize primary and secondary clinical outcome measures. To this end, the authors have collaborated across our respective trials of psilocybin-assisted psychotherapy for methamphetamine use disorder.

### 3.5. Trial design

There are numerous questions regarding optimal trial and treatment design across psilocybin-assisted psychotherapy trials for any indication, including the optimal therapeutic modality and number of preparatory and integrative psychotherapy sessions. Previous studies of psilocybin-assisted psychotherapy for other substance use disorders paired psilocybin with motivational enhancement therapy or addiction-focused cognitive behavioral therapy (30–32). A variety of other evidence-based non-pharmacological interventions for methamphetamine use disorder could also be investigated in combination with psilocybin administration (13).

Finally, treatment blinding continues to be a challenge for high-dose psychedelic trials due to the lack of an adequate active placebo to use as a comparator. Historically, drug regulators require results from studies in which a double-blinded control condition exists (and participants are randomized) in order to achieve registration and subsidy; thus, the focus has been on controlling for the drug component over the psychotherapy. Beyond the concerns of regulatory approval and coverage, trials could consider comparators more like those within psychotherapy trials, such as an established evidence-based treatment for methamphetamine use disorder, treatment-as-usual, or a waitlist control. However, uncertainty remains regarding the optimal treatment for methamphetamine use disorder, treatment-as-usual varies substantially across treatment settings, and use of a waitlist control can present ethical issues among high-risk clinical populations (89).

## 4. Summary

Methamphetamine use disorder is an increasing public health crisis (9) with limited treatment solutions. Above, we describe the rationale and impetus to support the investigation of psilocybin-assisted psychotherapy for the treatment of methamphetamine use disorder. A range of study design considerations specific to this indication, and collaboration among study teams to harmonize data collection in early-stage trials, will help ensure important questions are answered while balancing safety and access. These approaches will most efficiently inform larger trials in the future, should initial outcomes be promising, aimed at addressing the urgent need for better options to treat methamphetamine use disorder.

## Author contributions

JB and CSS conceptualized the manuscript and wrote the initial draft. All authors contributed to the manuscript and approved the submitted version.
